# A Case of Rapid Transformation of a Nail Matrix Nevi to Melanoma After Messenger RNA COVID-19 Vaccine: A Cause or a Coincidence?

**DOI:** 10.7759/cureus.76312

**Published:** 2024-12-24

**Authors:** Maria Farhat, Joseph Zouein, Jad Abou Khater, Anne-Sophie Sarkis, Josiane Helou

**Affiliations:** 1 Department of Dermatology, Saint Joseph University, Hôtel-Dieu de France Hospital, Beirut, LBN; 2 Department of Medicine, Duke University Medical Center, Durham, USA; 3 Department of Dermatology, Hôpital Erasme, Université Libre de Bruxelles, Brussels, BEL

**Keywords:** dermoscopy, longitudinal melanonychia, mrna-based vaccine, subungual melanoma, surveillance

## Abstract

Subungual melanoma is a variant of acral lentiginous melanoma that arises from the nail matrix. Subungual melanomas present unique clinical challenges due to diagnostic difficulties and the lack of a standardized protocol for surveillance, also, there are no evidence-based studies that determine the ideal frequency and duration of clinical and dermoscopy follow-ups in patients with longitudinal melanonychia. This is highlighted by a case of longitudinal melanonychia in a 53-year-old patient who underwent malignant transformation to subungual melanoma after a biphasic growth. Another problem raised is how long to observe longitudinal melanonychia, with the conclusion that it should be observed for lifetime. Finally, this report also illustrates the possible role of the COVID-19 messenger RNA vaccine in cancer development and/or progression.

## Introduction

Longitudinal melanonychia (LM) is a brown-to-black band extending from the matrix to the distal nail plate [1]. It results from an increased activation of melanocytes and/or melanocytic hyperplasia, leading to increased deposition of melanin pigment within the nail matrix.

Nail unit melanoma (NUM) or subungual melanoma (SUM) is a variant of acral lentiginous melanoma that arises from the nail matrix [2]. Although it is very rare and accounts for two to three percent of all cutaneous melanomas, it is the most common type of melanoma in deeply pigmented individuals [3].

Subungual melanomas present unique clinical challenges due to diagnostic difficulties resulting in delays in diagnosis or misdiagnosis, and also due to the lack of a standardized protocol for surveillance [4]. In fact, there are no evidence-based studies that determine the ideal frequency and duration of clinical and dermoscopic follow-ups in patients with longitudinal melanonychia of adult onset [4]. Due to these challenges, many subungual melanomas are identified at later stages and consequently have worse prognoses and survival outcomes compared to other cutaneous melanoma subtypes [4].

## Case presentation

We report hereby the case of a 53-year-old patient, with no pertinent personal nor familial medical history, who consulted our clinic in May 2023 for a rapidly growing and darkening melanonychia of the left thumb.

She previously consulted our clinic in mid-2018 for a longitudinal melanonychia of the left thumb that she had noticed two years prior to her visit. In fact, she recalled the presence of a brownish-black dot over the cuticle of the same thumb 10 years ago that was stable all along. A watch-and-wait strategy was chosen, and she was advised to come back for follow-up in six months, and earlier in case of rapid growth or change in aspect or color.

**Figure 1:**
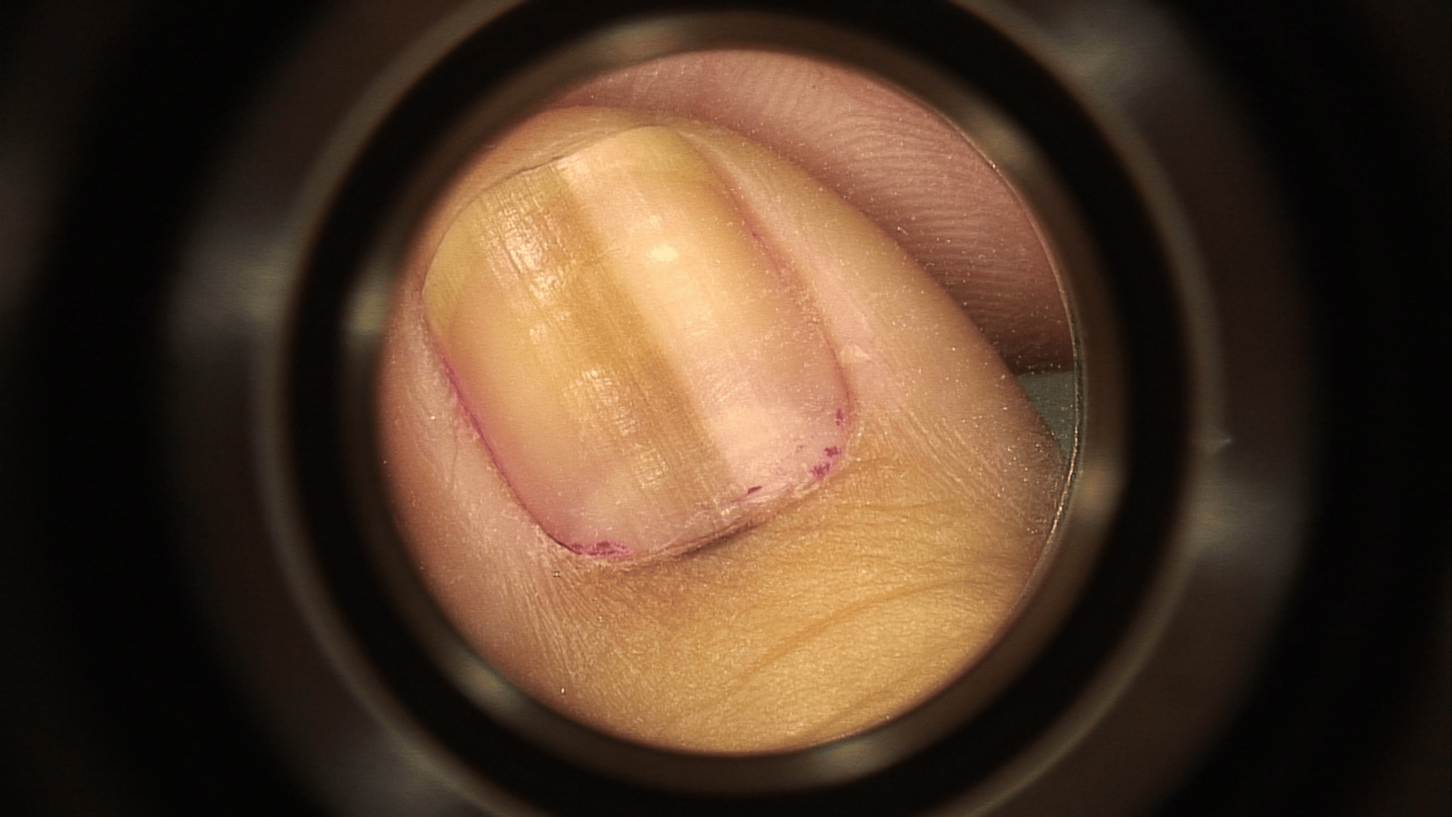
Dermoscopic image of the melanonychia after the first slow growth. A micro-Hutchinson sign is noted.

She consulted again in 2019, where the melanonychia slightly progressed in size (less than 3 mm) with slightly variable-sized bands and different shades of brown. A micro-Hutchinson sign was noted. Clinical and dermoscopic images (Figure 1) were taken, and the patient was advised to come back in three months.

**Figure 2:**
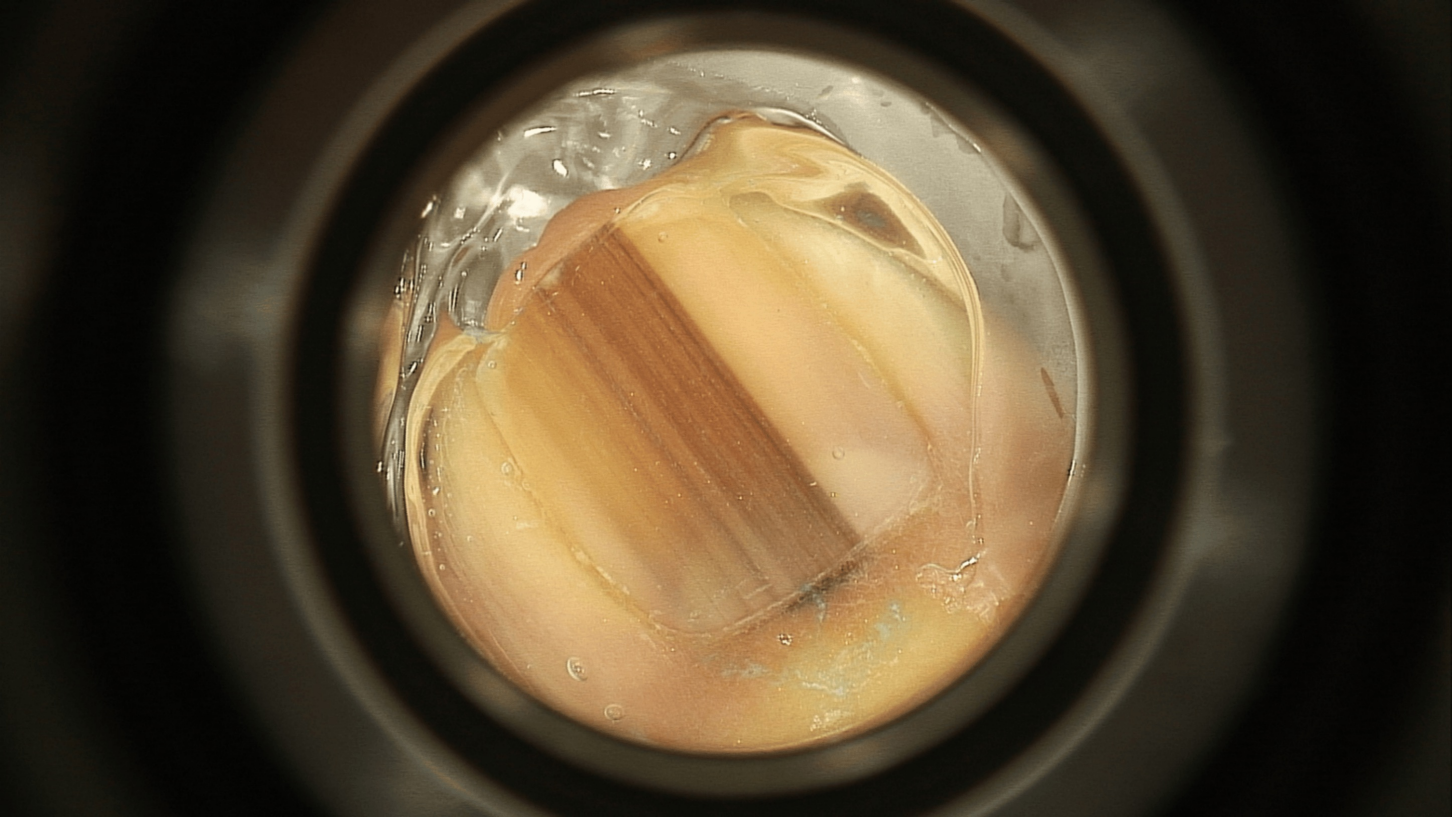
Dermoscopic image of the melanonychia after the second rapid growth and malignant transformation. Note the heterogeneous longitudinal black-brown lines of irregular thickness and the positive Hutchinson sign.

She was lost to follow up for three years, until she came back in May 2023 for recent darkening and widening (>5 mm) of the melanonychia that developed rapidly over the last three to five months, with heterogeneous longitudinal black-brown lines of irregular thickness and a positive Hutchinson sign (Figure 2).

**Figure 3:**
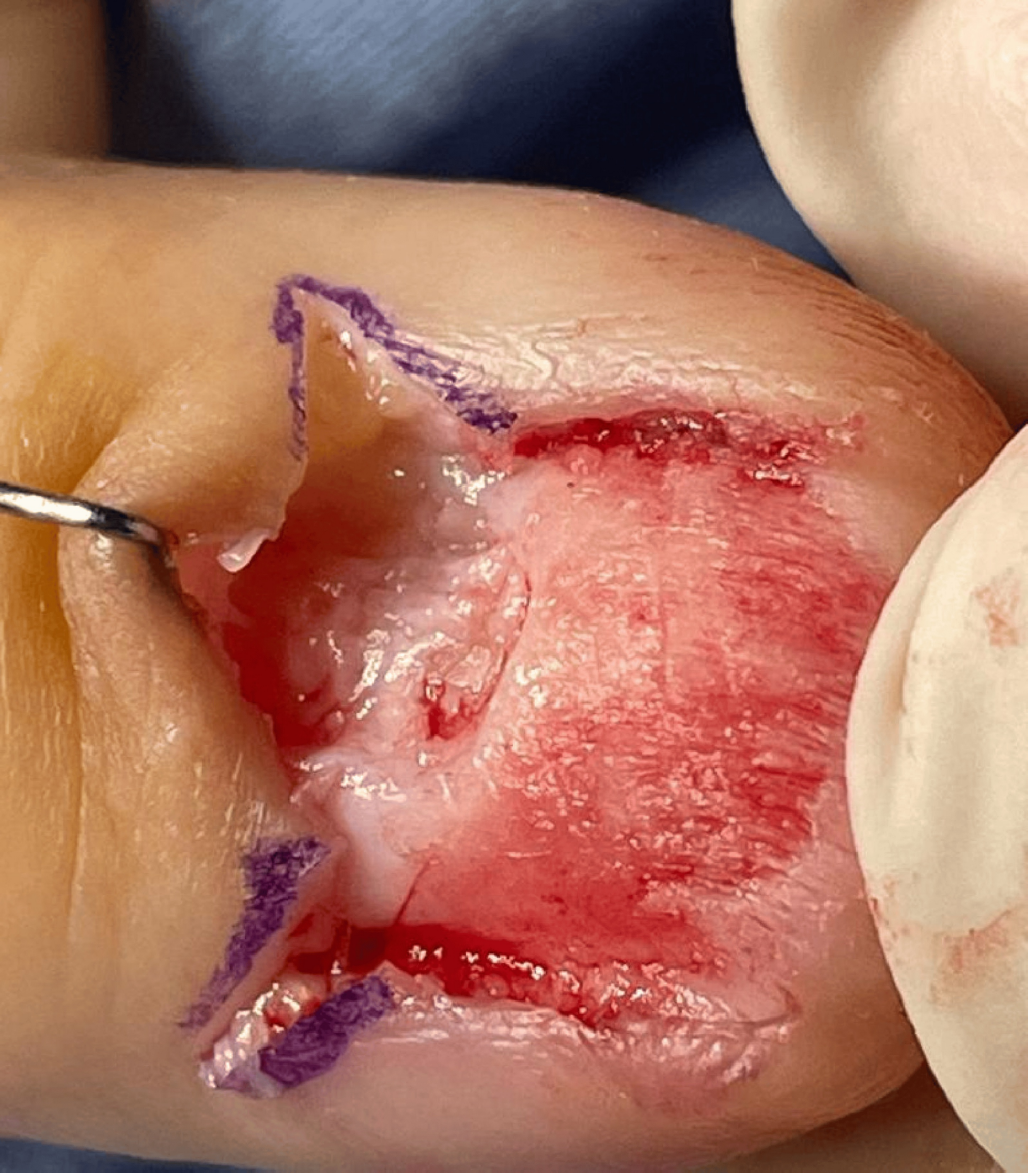
Surgical excision of the melanonychia

An excisional biopsy (Figure 3) revealed the diagnosis of an in-situ acral lentiginous melanoma of the left thumb. Of note, the patient has a Fitzpatrick skin type III and she has no known risk factors for melanoma development such as the family history of melanoma or the use of ultraviolet nail lamps.

Moreover, the patient mentioned that between 2021 and 2023, before the rapid growth of the melanonychia, she had received three doses of mRNA COVID-19 vaccine.

## Discussion

Our case illustrates a malignant transformation of an apparently benign longitudinal melanonychia following the administration of three doses of mRNA COVID-19 vaccines.

The pathophysiology of subungual melanoma is not completely elucidated. Its pathogenic triggers may include ultraviolet radiation, chemical burns, malignant changes of poor tissue structure, external trauma, metabolic deficiencies, pigmentation, endocrine disorders and others [5]. The symptoms of the disease are not obvious at first and patients tend to ignore it as a mole or pigmentation [5].

Consensus regarding the frequency and duration of follow-ups of pigmented nail lesions lacks. One suggestion is to monitor the patient every six months with dermoscopy in cases of benign melanocytic lesions and to have a strict follow-up every three months in cases of irregular pigmentation without other signs of malignancy, but clinicians should be aware that changes may present earlier [6].

Dermoscopy should be used routinely in the evaluation of a pigmented nail, as it provides important information for the management of melanonychia and can help avoid unnecessary nail biopsies with resultant nail dystrophy [7]. Our case suggests that prolonged follow-up is mandatory for early detection of possible malignant changes.

Ungual melanomas are rare, and there are controversies in their diagnosis regarding the duration of surveillance and whether to undergo a nail matrix biopsy or a complete excision when a malignant transformation is suspected [8]. Regarding this case, if we had biopsied the patient during the first growth and the pathology came back negative, during the second rapid growth, the result of the first biopsy cannot be assumed, and relying on it might delay the diagnosis of melanoma. The debate is whether to re-biopsy the lesion or to totally excise it.

The mRNA COVID-19 vaccine might be incriminated in the rapid growth of the lesion. The patient had received three doses of the mRNA vaccine before her last visit. These vaccines were shown to inhibit essential immunological pathways [9]. In fact, adding 100% of N1-methyl-pseudouridine to the mRNA vaccine in a melanoma model was shown to stimulate cancer growth and metastasis, while non-modified mRNA vaccines induced opposite results, thus suggesting that COVID-19 mRNA vaccines could aid cancer development [9]. Due to the novel mRNA technology used in COVID-19 vaccines, long-term relatively rare adverse events are yet to be assessed [10].

## Conclusions

In conclusion, patients should be instructed to consult when any change is noted in longitudinal melanonychia. Detecting a nail unit melanoma at an early stage greatly increases the chances of survival.

Longitudinal melanonychia should be observed for a long time, as malignant transformation could happen years later and precise guidelines regarding the frequency and duration of follow-ups must be established.

Furthermore, future research may elucidate any potential association between novel vaccines such as the mRNA COVID-19 vaccine and cancer development or progression.
